# Fishing line assisted endoscopic placement of multiple plastic biliary stents for unresectable malignant hilar biliary obstruction: a retrospective study

**DOI:** 10.1186/s12876-021-02014-x

**Published:** 2021-11-19

**Authors:** Huahui Zhang, Fengdong Li, Jian Huang, Chunyan Huo, Jin Huang

**Affiliations:** 1 Graduate School of Dalian Medical University, Dalian, China; 2 Department of Gastroenterology, The Affiliated Changzhou No.2 People’s Hospital of Nanjing Medical University, No. 68, Gehu Middle Road, Wujin District, Changzhou City, 213100 Jiangsu Province China

**Keywords:** Plastic biliary stent, Fishing line assisted method, Stent migration, Endoscopic retrograde cholangiography, Malignant hilar biliary obstruction

## Abstract

**Background and aims:**

Stent migration is one of the most common complications during the placement of multiple plastic biliary stents (MPBS) under endoscopy. This study aims to evaluate the feasibility and efficiency of the fishing line assisted (FLA) method for preventing the complication.

**Methods:**

Patients with unresectable malignant hilar biliary obstruction (MHBO) who undergone endoscopic placement of MPBS using the FLA or conventional method from May 2018 to April 2021 in our center were enrolled in the study. The endpoints of this study were the stent migration rate, technical success rates, adverse events rates, times of stent migration, and the procedure time.

**Results:**

FLA group (N = 19) and conventional group (N = 22) had similar baseline characteristics of the patients. The technical success rates (100% vs. 95.5%; *P *> 0.05), ERCP-related adverse events rates (5.3% vs. 4.5%; *P *> 0.05), and the stent-related adverse events rates (0% vs. 4.5%; *P* > 0.05) were no significant differences between the FLA and conventional groups. MPBS inserted using the conventional method consumed more time (median, 33.9 min vs. 15.6 min; *P *< 0.05) method and increased the times of stent migration (median, 3 times vs. 0 times; *P* < 0.05) than using the FLA method. Even if no statistical difference was detected in the stent migration rate between groups, this rate was lower in the FLA group than the conventional group (0% vs. 13.6%; *P *> 0.05).

**Conclusions:**

FLA method is an effective technique for MPBS implantation to prevent stent migration during endoscopic retrograde cholangiography (ERCP). The method should be applied to patients with unresectable MHBO who need to place MPBS.

**Supplementary Information:**

The online version contains supplementary material available at 10.1186/s12876-021-02014-x.

## Background

MHBO results from cholangiocarcinoma, hepatocellular carcinoma, gallbladder cancer, and other cancer metastasis to the regional lymph nodes [1, 2]. Patients with MHBO have a poor quality of life owing to pruritus, loss of appetite, and general debility caused by jaundice  [3, 4]. Biliary drainage is widely used to relieve those symptoms in patients with MHBO and improve the quality of life of patients. The placement of stents during ERCP in the obstruction region is an effective palliation method, this program is often done by the experienced endoscopist [5, 6]. Plastic biliary stent (PBS) placement has been firstly reported in the early 1980s [7], PBS is more economical and the effect is similar [8], compared with metal stents. Patients with unresectable MHBO usually need to insert MPBS during ERCP [9–11]. However, the first stent upward migration usually occurred during the second stent inserted due to the friction between the two stents. Endoscopists had to spend a lot of time adjusting the position of the stents. Based on the previous literature data, many methods were reported to prevent stent upward and downward migration after stents implantation rather than the process of stents implantation [12–14]. To fill the gap in this problem, we invented a novel technology known as the FLA method. In the study, we evaluate the safety and feasibility of the method for MPBS placement.

## Methods

### Patients

In total, consecutive 41 patients from May 2018 to April 2021 in our center meet our inclusion criteria. The inclusion criteria for the study were as follows: (1) patients who had unresectable MHBO and (2) patients who performed endoscopic biliary drainage using MPBS by the FLA or conventional method. (3) All clinical, laboratory, and imaging data of the patients can be obtained from our medical record system. The exclusion criteria for this research were as follows : (1) patients had a history of both biliary stent placement and percutaneous transhepatic biliary drainage (PTBD). (2) Two stents were both inserted into the right or left hepatic duct and (3) clinical data of patients is incomplete. The location of obstruction was based on examination of computer tomography (CT), magnetic resonance imaging (MRI), or ERCP. Malignant stenosis involving the hilum is classified by using the Bismuth–Corlette classification system. Bismuth type I stenosis involves the proximal common hepatic duct and spares the confluence between the left and right ductal systems. Type II stenosis involves the confluence and spares the segmental hepatic ducts. Type IIIa and IIIb stenosis involve either the right or left segmental hepatic duct, respectively, and type IV stenosis involves the confluence and both the right and left segmental hepatic ducts. All patients had no contradiction for endoscopic biliary drainage and gave written informed consent for the ERCP procedure.

### Stents

**Fig. 1:**
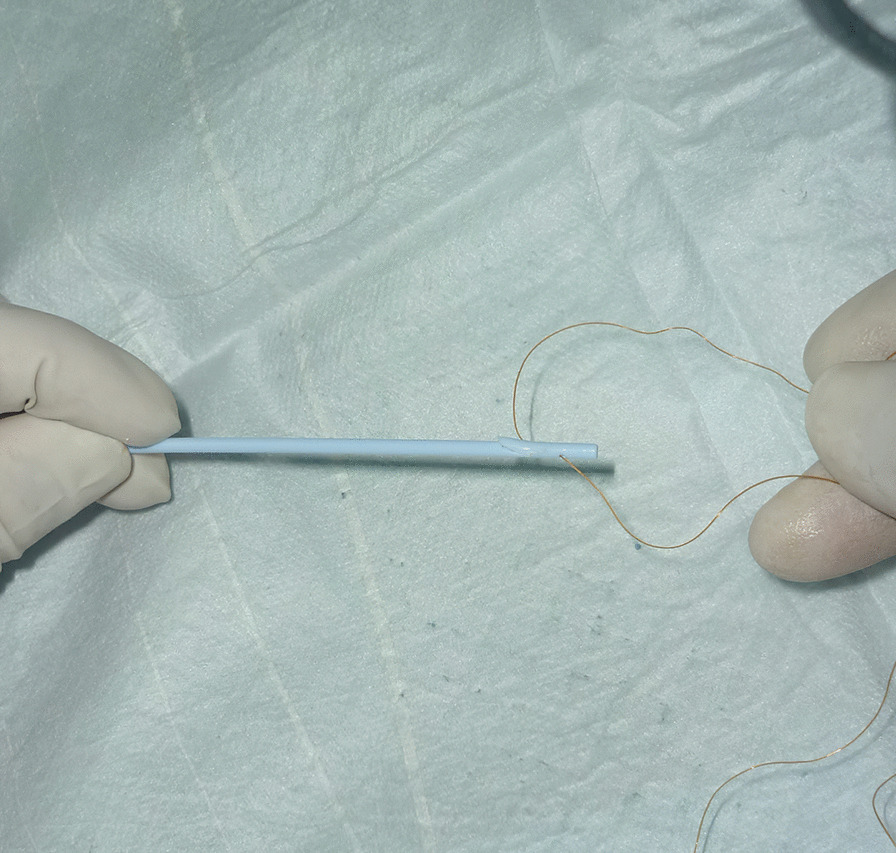
The fishing line was going through the side hole at the distal end of the first PBS and two endpoints of the fishing line were aligned (Before ERCP procedures)

Straight stents with a diameter of 7-Fr or 8.5-Fr were used in our study. We created two side holes of the distal end of the first plastic stent using a scalpel on a sterile operating table which was to make sure the fishing line going through (Fig. 1).

### FLA method for stent implantation

**Fig. 2:**
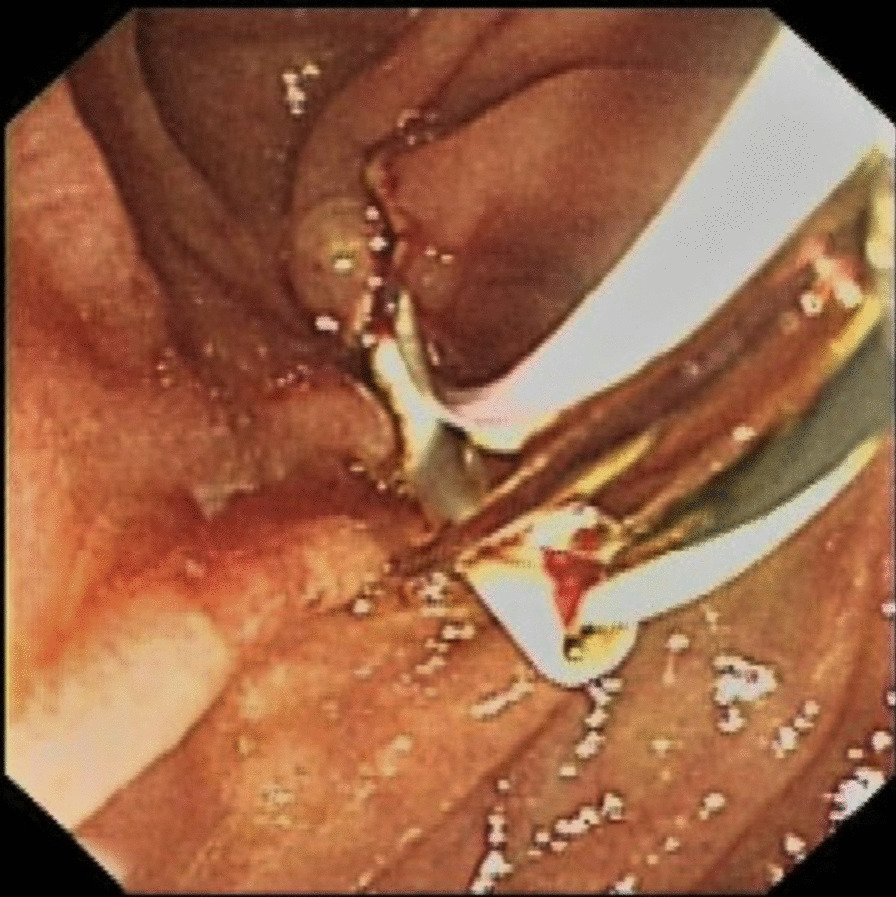
The first PBS was inserted into the right or left hepatic duct through the endoscopic channel with the assistance of a stent delivery catheter, meanwhile, the fishing line at the distal end of the first PBS could be observed under endoscopy

**Fig. 3:**
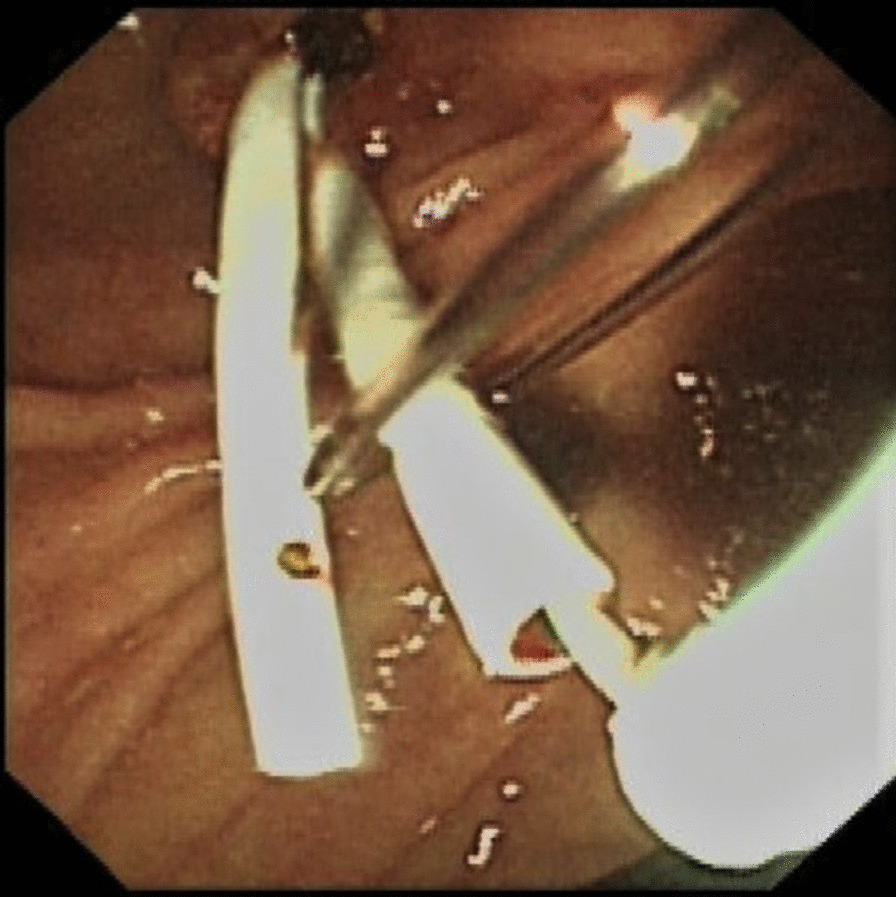
The second PBS was placed in the other side hepatic duct, meanwhile, the fishing line maintained tension to prevent the first PBS upward migration

**Fig. 4:**
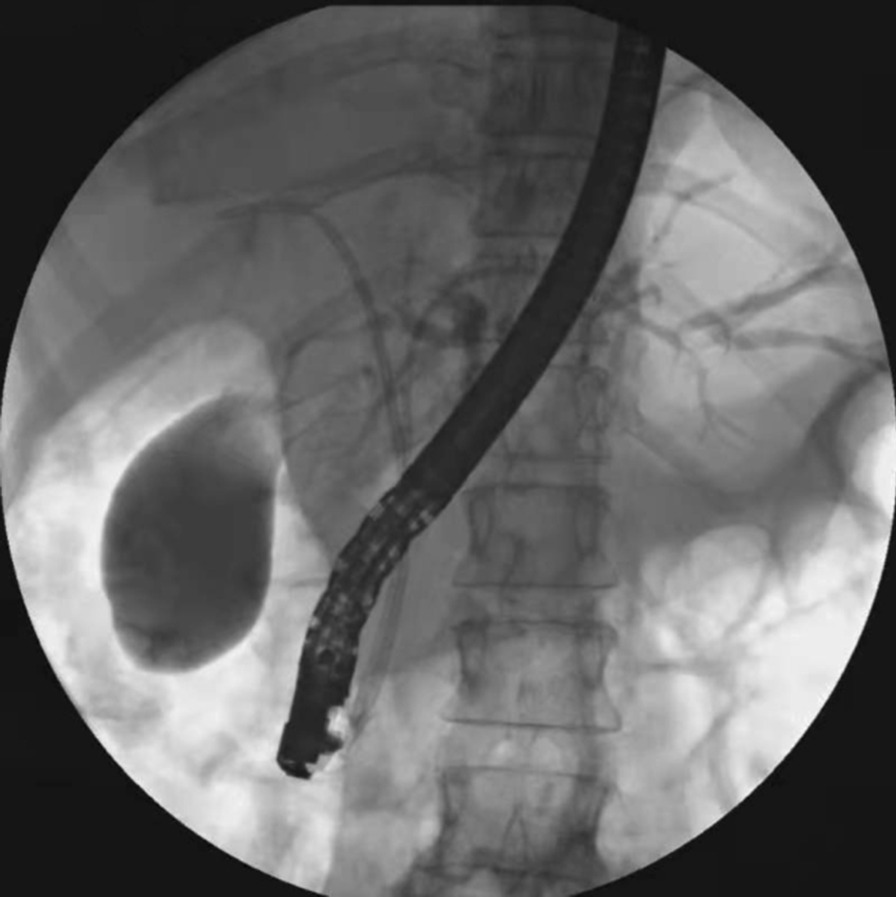
Two PBS were successfully implanted in the left and right hepatic duct

All patients underwent stent implantation under anesthesia and sedation in our endoscopic operating room. ERCP was performed in all patients with a gastroduodenal endoscope by two senior experienced endoscopists before the stent implantation. After successful bile duct intubation, the most severe part of the bile duct dilatation in right or left hepatic duct was inserted a guide wire and the structure was dilated repeatedly by ballon under the X-ray fluoroscopy guidance, respectively. Before the implantation of the stent, a sphincterotomy was performed to facilitate the implantation of stents. The stent placement inside the bile duct was as follows: (1) After being treated with liquid paraffin, a 3-m long fishing line was going through the side hole at the distal end of the first PBS (Fig. 1). (2) The first PBS was inserted into the right or left hepatic duct along the guidewire through the endoscopic channel using a stent delivery catheter. And the double tails of the fishing line were left outside of the endoscopic channel for hand control (Fig. 2). (3) The first PBS was pulled by the fishing line and kept its desired location under endoscopic and fluoroscopic surveillance when the second PBS was inserted into the other side hepatic duct. Besides, the fishing line always maintained tension to prevent the first PBS upward migration during the process of the second PBS implantation (Fig. 3). (4) After the second stent was placed successfully the clever knife was used as a stent delivery catheter to withstand the distal end of the first PBS, then either endpoint of the fishing line was grabbed at the entrance to the endoscopic channel and the fishing line was pulled out of side hole at the distal end of the first PBS through the endoscopic channel, at last, the guidewire was withdrawn from the stents (Additional file 1: Video 1) (Fig. 4).

### Conventional method for stent implantation

After successful bile duct cannulation, guidewires were led to the left hilar duct and/or the right hilar duct under the surveillance of fluoroscopy. Similar to the FLA method the structure was also dilated by ballon and a sphincterotomy was performed. At last, the first and second stents were inserted into the bile duct along guidewires using a stent delivery catheter. The appropriate length of prostheses was selected according to the location of strictures. At the 3 months after procedures, ERCP was performed for stent exchange or removal.

### Outcomes

The stent migration rate, technical success rates, times of stent migration, adverse events, and procedures time between two groups were evaluated in this study. Proximal migration was defined as when stent was observed in the duct on fluoroscopy and the distal end of the stent was not observed under endoscopy. Distal migration was defined as when stent located below the original position or was not observed under either endoscopy or fluoroscopy. Technical success was defined as the placement of all stents over the stricture location, the distal end of the stent in the intestinal cavity is almost flush and constraint medium or bile juice can flow the stent. The procedure time was recorded from the preparation of implantation of the first stent to the second stent was inserted completely. The times of stent migration were defined as the number of times the first stent migrates into the bile duct or duodenal intestinal cavity during ERCP. Cholangitis, pancreatitis, bleeding, and perforation were recorded as ERCP-related adverse events. Stent-related adverse events included biliary bleeding and perforation.

### Statistics

Continuous data conforming to the normal distribution are represented as mean ± standard deviation (SD), or continuous data are represented as median (range). Categorical variables were compared with the Chi-square test or Fisher’s exact test. Continuous variables were compared with by Student’s or non-parametric Wilcoxon rank-sum test. A *P *< 0.05 was considered to be statistically significant. All data processing and analysis were used by IBM SPSS.

## Results

### Patients information

There were 41 patients were enrolled in our research. 19 patients in the FLA group and 22 patients in the conventional group were analyzed in our study. Two groups had similar baseline characteristics (Table 1).

**Table 1:** Characteristics of the patients

	FLA group (N = 19)	Conventional group (N = 22)	*P* value
Sex (Male/Female)	11/8	13/9	0.94
Age (median, years) (range)	68 (52–82)	70 (56–88)	0.21
Clinical symptoms, n (%)			
Jaundice	11 (57.9)	12 (54.5)	0.83
Abdominal pain	10 (52.6)	9 (40.9)	0.45
Weight loss	8 (42.1)	11 (50)	0.61
Anorexia	7 (36.8)	6 (27.3)	0.51
Fatigue	3 (15.8)	5 (22.7)	0.87
Laboratory data			
T-Bil (median, mg/dL) (range)	6.3 (2.8-13.1)	5.8 (3.6–17.5)	0.65
Albumin (mean±SD, g/dl)	3.3 ± 0.72	3.5 ± 0.60	0.59
ALT (median, IU/L) (range)	56.8 (10.5–1319.0)	60.4 (12.9–789.8)	0.43
CA19-9 (median, U/mL) (range)	110.2 (12.6–336.7)	97.7 (17.6–446.7)	0.11
Causes of strictures, n (%)			0.97
Hilar cholangiocarcinoma	8 (42.1)	10 (45.5)	
Gallbladder carcinoma	4 (21.1)	5 (22.7)	
Hepatocellular carcinoma	3 (15.8)	4 (18.2)	
Metastatic cancers	4 (21.1)	3 (13.6)	
Bismuth classification (%)			0.88
II	2 (10.5)	1 (4.5)	
III	4 (21.1)	5 (22.7)	
IV	13 (68.4)	16 (72.7)	
TNM stage, n (%)			0.86
II	2 (10.5)	2 (9.1)	
III	3 (15.8)	2 (9.1)	
IV	14 (73.7)	18 (81.8)	
Basic diseases, n (%)			
Hypertension	5 (26.3)	6 (27.3)	0.95
Diabetes	2 (10.5)	2 (9.1)	0.88
Cardiovascular disease	3 (15.8)	3 (13.6)	0.85
Kidney disease	1 (5.3)	3 (13.6)	0.71
Stent size			0.76
7Fr+7Fr	9 (47.4)	13 (59.1)	
8.5Fr+8.5Fr	7 (36.8)	6 (27.3)	
7Fr+8.5Fr	3 (15.8)	3 (13.6)	

### Technical success rates, the stent migration rate, and times of stent migration

All patients were successfully inserted MPBS in the FLA group. The technical success rates were 95.5% (21/22) in the conventional group. The technical success rates were no difference between the two groups. One patient (4.5%) in the conventional group had failed MPBS inserted owing to longer procedure time, and the patient was performed MPBS placement successfully using the FLA method. A total of 3 patients (13.6%) experienced stent migration in the conventional group, proximal migration in two cases (9.1%) and distal migration in one case (4.5%). None of the patients experienced stent migration in the FLA group. All migrated stents were retrieved under endoscopy. There was no significant difference in the stent migration rate between groups (0% VS 13.6%; *P *> 0.05). The median (range) times of stent migration were 0 (0–2) and 3 (1–5) times in the FLA and conventional groups, respectively. The times of stent migration between the FLA and conventional groups were statistically significant (median, 0 times vs. 3 times; *P *< 0.05) (Table 2).

**Table 2:** Outcomes

	FLA group (N = 19)	Conventional group (N = 22)	*P* value
The stent migration rate, n (%)	0 (0)	3 (13.6)	0.10
Technical success rates, n (%)	19 (100)	21 (95.5)	0.35
Procedures time, (median, min) (range)	15.6 (12.6–23.3)	33.9 (21.8–38.7)	0.00
Adverse events, n (%)			
ERCP-related	1 (5.3)	1 (4.5)	0.92
Pancreatitis	1 (5.3)	1 (4.5)	0.92
Others	0 (0)	0 (0)	–
Stent-related	0 (0)	1 (4.5)	0.35
Biliary perforation	0 (0)	0 (0)	–
Biliary bleeding	0 (0)	1 (4.5)	0.35
Times of stent migration,(median, times) (range)	0 (0-2)	3 (1-5)	0.00

### Adverse events and procedures time

One patient (5.3%) in the FLA group and one patient (4.5%) in the conventional group experienced mild pancreatitis after the procedure and recovered shortly after treatment. None patients developed biliary bleeding or perforation in the FLA group. One patient (4.5%) experienced biliary bleeding in the conventional group. The median time of procedures was 33.9 min ranged from 21.8 to 38.7 min in the conventional group. The procedures time ranged from 12.6 to 23.3 min with a median time of 15.6 min. The time of procedures in the conventional group was significantly longer than the FLA group (median, 33.9 min vs. 15.6 min; *P *< 0.05) (Table 2).

## Discussion

ERCP and PTBD are the most common technology for biliary drainage in MHBO. However, PTBD drains bile out of the body which may disrupt the patient’s digestive function. A study in the United States showed that patients performed by ERCP experienced fewer adverse events than patients who performed PTBD [15]. A recent randomized clinical trial study had to terminate because of the high mortality rate in the PTBD group. Their existing data also shows that ERCP was safer than PTBD [16]. Therefore, ERCP usually was the first choice for biliary drainage in MHBO. However, the first stent tends to migrate to the bile duct, and the second stent incline to migrate to the duodenum during ERCP. We created a novel method named the FLA method to solve the problem. Our study indicates that the FLA method can effectively assist the placement of the bile duct stent, and reduce the procedure time (median, 15.6 min vs. 33.9 min; *P *< 0.05) and the times of stent migration (median, 0 times vs. 3 times; *P *< 0.05) compared with the conventional method.

Friction between the bile duct and stents can’t be eliminated, especially in the case of bile duct stricture. On one hand, stents can stay close together due to friction between stents, on the other hand, stents were easy to migrate during stents inserted. Endoscopists need a lot of time to adjust the location of stents to make sure the biliary stents were located in the best position. The prolonged operation may increase the chance of cardiovascular and cerebrovascular complications. Repeatedly adjusting the position of the stent in the bile duct may result in bile duct damage. In the conventional group, one patient experienced biliary bleeding. It may be due to the long-time friction between the stent and bile duct.

A Japanese study reported a modified PBS for hilar stenosis [11]. A Nylon thread attached to a hole punched into the distal end of the stent for removal. In this study, the stent was implanted above the papilla of the duodenum, which was called “inside stent” [11, 17]. However, there was no obvious evidence that the inside stent could prevent the first stent migration during the second stent implantation. Besides, migration or dislocation of stents was still a problem for inside stent [18]. Furthermore, a randomized trial study reported that the stent was difficult to remove in the inside stent group [19]. The duodenal papilla and bile duct may be damaged during the process of retrieving the stents. Single pigtail stent may have prevented the stent upward migration, however, single pigtail stent was expensive, and it may be easy to migrate to the duodenum owing to the pigtail structure. Besides, single pigtail stent was widely used to implant the pancreatic duct rather than the bile duct. We developed a technique named the “FLA method” in the endoscopic placement of MPBS. In this study, compared with conventional techniques, the FLA method for MPBS implantation, not only minimizes the consumption of time but also reduces the rate of stent migration. The overall stent migration rate was 7.3% (3/41) in the current study. The incidence of stent migration had been reported to range from 5 to 10% in biliary stenting patients [14, 20, 21], which was similar to our results. The stent migration rate in the FLA group was lower than that in the conventional group, but it was not statistically significant (0% VS 13.6%; *P *> 0.05). The reason may be that the sample of our study was too small. The merit of this technique is below: (1) the first stent was pulled and fixed by the fishing line. At the time of subsequent stents insertion, the fishing line could work as the anchor by grabbing its endpoints to avoid the previous stents migrating to the proximal bile duct. (2) Guidewires were left in stents over the process to adjust their position expediently. If the first stent migrated down to the duodenum, we inserted a knife along the guidewire to push the stent to the desired position under the monitoring of X-ray with fishing line release. (3) While the fishing line was pulled out of the side hole in the first stent, the knife was inserted along the guidewire and withstand the endpoint of the stent to maintain its location.

There are some limitations to this study. First, the sample size was small a large-scale multicenter study is required to meet the statistical calculation. A second limitation is the lack of blinding of the physician to the procedure.

In conclusion, the FLA method is an effective technique for multiple plastic biliary stents implantations to prevent stent dislocation. It improves the accuracy of the stent’s location and reduces the need for time-consuming.

## Supplementary Information

**Additional file 1: Video 1**. It shows the process of retrieving the fishing line from the first PBS. In brief, either endpoint of the fishing line was grabbed and pulled at the entrance to the endoscopic channel to remove the fishing line from the side hole of the first PBS.

## Data Availability

The datasets used or analyzed during the current study are available from the corresponding author on reasonable request.

## Abbreviations

MPBS: Multiple plastic biliary stents
FLA: Fishing line assisted
MHBO: Malignant hilar biliary obstruction
ERCP: Endoscopic retrograde cholangiography
PBS: Plastic biliary stent
PTBD: Percutaneous transhepatic biliary drainage
CT: Computer tomography
MRI: Magnetic resonance imaging
SD: Standard deviation
T-Bil: Total bilirubin
ALT: Alanine aminotransferase
